# Catalytic Mode and Product Specificity of an α-Agarase Reveal Its Direct Catalysis for the Production of Agarooligosaccharides

**DOI:** 10.3390/foods13152351

**Published:** 2024-07-26

**Authors:** Xiaofeng Zeng, Yixiong Tian, Haocun Kong, Zhaofeng Li, Zhengbiao Gu, Caiming Li, Yan Hong, Li Cheng, Xiaofeng Ban

**Affiliations:** 1 School of Food Science and Technology, Jiangnan University, Wuxi 214122, China; 6210113132@stu.jiangnan.edu.cn (X.Z.); yixiongtian@jiangnan.edu.cn (Y.T.); haocunkong@jiangnan.edu.cn (H.K.); zfli@jiangnan.edu.cn (Z.L.); zhengbiaogu@jiangnan.edu.cn (Z.G.); licaiming2009@126.com (C.L.); hongyan@jiangnan.edu.cn (Y.H.); chenglichocolate@163.com (L.C.); 2 State Key Laboratory of Food Science and Resources, Jiangnan University, Wuxi 214122, China

**Keywords:** α-agarase, catalytic mode, product specificity, agarooligosaccharides

## Abstract

Many α-agarases have been characterized and are utilized for producing agarooligosaccharides through the degradation of agar and agarose, which are considered valuable for applications in the food and medicine industries. However, the catalytic mechanism and product transformation process of α-agarase remain unclear, limiting further enzyme engineering for industrial applications. In this study, an α-agarase from *Catenovulum maritimus* STB14 (Cm-AGA) was employed to degrade agarose oligosaccharides (AGOs) with varying degrees of polymerization (DPs) to investigate the catalytic mechanism of α-agarases. The results demonstrated that Cm-AGA could degrade agarose into agarotetraose and agarohexaose. The reducing ends of agarotetraose and agarohexaose spontaneously release unstable 3,6-anhydro-α-l-galactose molecules, which were further degraded into agarotriose and agaropentose. Cm-AGA cannot act on α-1,3-glucoside bonds in agarotriose, agarotetraose, neoagarobiose, and neoagarotetraose but can act on AGOs with a DP greater than four. The product analysis was further verified by β-galactosidase hydrolysis, which specifically cleaves the non-reducing glycosidic bond of agarooligosaccharides. Multiple sequence alignment results showed that two conserved residues, Asp994 and Glu1129, were proposed as catalytic residues and were further identified by site-directed mutagenesis. Molecular docking of Cm-AGA with agaroheptose revealed the potential substrate binding mode of the α-agarase. These findings enhance the understanding of Cm-AGA’s catalytic mode and could guide enzyme engineering for modulating the production of agarooligosaccharides.

## 1. Introduction

Agar is the main linear polysaccharide present in cell walls and is produced by red seaweeds (*Rhodophyceae*) [1] wherein agarose plays the primary role in the gel-forming process of agar. It is a chain polymer comprising β-D-galactose (D-Gal) and 3,6-anhydro-α-l-galactose (L-AHG) linked through alternating α-1,3 and β-1,4 glycosidic bonds [2,3]. Agarose can be degraded into several agarose oligosaccharides (AGOs) [4], including agarooligosaccharides (AOSs) having D-Gal as the non-reducing end and neoagarooligosaccharides (NAOSs) having L-AHG as the non-reducing end [5]. AGOs have various physiological activities, such as prebiotic [6], antioxidant [7], anti-inflammatory [8], hepatoprotective [9], skin-whitening [10], and potential protective effects against neurotoxicity [11]. Due to these activities, AGOs have extensive latent applications in the food, cosmetic, and pharmaceutical industries. Chemical or biological degradation of agarose can lead to AGO production [12]. Therein, compared with chemical methods, the enzymatic degradation method of agarases is efficient and environmentally friendly and achieves the directed production of AGOs. Thus, agarases have received extensive research attention [13].

Numerous agarases from marine and terrestrial bacteria have been characterized [14,15,16,17]. Based on the differences in the catalytic mode, agarases are classified as α-agarases (EC 3.2.1.158) [18] and β-agarases (EC 3.2.1.81) [19]. α-Agarases can specifically cleave α-1,3 linkages in agarose to produce AOSs, while β-agarases can specifically cleave β-1,4 linkages in agarose to produce NAOSs. Despite years of research, only nine α-agarases [20] have been characterized to date. All the nine α-agarases belong to the glycoside hydrolase family 96 (GH96 family) [19], determined on the basis of the multiple sequence alignment. Identifying an α-agarase is difficult because this enzyme has a relatively high molecule mass and a complex structure with low stability, which makes heterologous expression and isolation of this enzyme challenging [21]. Moreover, the substrate-binding mode, catalytic mechanism, and product specificity-modulating molecular mechanism of agarase remain unclear. Therefore, exploring the catalytic mode and product specificity of Cm-AGA can offer a basis for the directed production and functional study of AOS.

Based on previous studies [20], we here successfully expressed an α-agarase from the marine bacterium *Catenovulum maritimum* STB14 (Cm-AGA) in *Escherichia coli* BL21(DE3). The Cm-AGA is the first reported α-agarase with agarobiose and agarotetraose as the primary catalytic products, indicating its significant potential in the production of low-molecular-weight AGOs. Through multiple sequence alignment, Cm-AGA has been identified as belonging to the GH96 family. Utilizing Cm-AGA to degrade agarose and analyzing the resulting enzymatic hydrolysates can lay the groundwork for functional studies of AGOs. Furthermore, the catalytic center and substrate-binding mode of Cm-AGA have been analyzed and verified, offering valuable insights into the structure-activity relationship of agarases and providing a theoretical basis for the targeted production of AOSs.

## 2. Materials and Methods

### 2.1. Gene Construction, Protein Production, and Purification

*E. coli* JM109, *E. coli* BL21(DE3), and plasmid pET-28a(+) were stored in our laboratory. pET-28a(+)*/Cm-aga* was constructed by inserting the *Cm-aga* gene (GenBank: MH005820.1) into the *NcoI* and *XhoI* sites of the pET-28a(+) vector. Then, after sequencing, the plasmid pET-28a(+)/*Cm-aga* was transformed into *E. coli* BL21(DE3) for enzyme production. A single colony was inoculated into a 100 μg/mL kanamycin-containing Luria–Bertani liquid medium and incubated at 37 °C until the OD_600_ reached 0.6–0.8. Subsequently, approximately 4% culture was transferred into 100 μg/mL kanamycin-containing terrific broth medium and incubated at 37 °C for 3 h until the OD_600_ reached 0.6–0.8. Isopropyl-β-D-thiogalactopyranoside was then added to the culture at a final concentration of 0.01 mM, and the culture was further incubated at 25 °C for 20 h for protein production [20]. The culture medium was centrifuged at 10,000× *g* for 20 min at 4°C. The harvested cells were resuspended in a lysis buffer (20 mM Tris-HCl buffer, pH 8.0) and sonicated in an ice bath by using an ultrasonic cell disruptor (Scientz Biotechnology, Ningbo, China). The suspension was centrifuged at 10,000× *g* for 10 min, and the crude Cm-AGA was obtained from the supernatant.

The recombinant protein was purified through Ni^2+^-affinity chromatography by using a HisTrap HP column (5 mL, GE Healthcare, Milwaukee, WI, USA). Buffer A (20 mM imidazole, 50 mM Tris-HCl, and 500 mM NaCl, pH 8.0) was applied to the column to equilibrium. The crude enzyme was applied through a 0.45-μm Millipore filter and loaded onto the column. The recombinant Cm-AGA was eluted with 80% buffer A and 20% buffer B (500 mM imidazole, 50 mM Tris-HCl, and 500 mM NaCl, pH 8.0). All steps were performed at 4 °C. The eluted protein was concentrated and desalted using a 50 kDa ultrafiltration centrifuge tube (15 mL, Millipore, Billerica, MA, USA) with 20 mM Tris-HCl buffer (pH 8.0). The protein purity and molecular mass were analyzed through sodium dodecyl sulfate–polyacrylamide gel electrophoresis (SDS-PAGE). Cm-AGA protein concentrations were determined using the Bradford method [22] with a Bradford Protein Assay Kit (Beyotime Biotechnology, Shanghai, China). The molar concentration was calculated by dividing the mass concentration by the molecular mass. The purified enzyme was stored at −80 °C for further analysis.

### 2.2. Activity Assay

The Cm-AGA activity was measured using the DNS method [23], with minor modifications. The substrate was agarose dissolved in Tris-HCl buffer (pH 8.0, 20 mM) at a concentration of 0.25% (*w*/*v*). The enzyme (100 μL) was reacted with 900 μL substrate by incubating them at 40 °C for 30 min. The reaction was halted by adding 1 mL of the DNS reagent and boiling the mixture for 5 min. After the samples were cooled, they were diluted with 2 mL of deionized water. The absorbance of the reducing sugar product was measured at 540 nm. A standard curve was prepared with D-Gal (0–0.8 mg/mL). One unit (U) of Cm-AGA activity was defined as the amount of enzyme required to produce 1 μmol of reducing sugar per minute [24] under the aforementioned reaction conditions. The solution without enzyme was used as a control.

### 2.3. SDS-PAGE Analysis

The intracellular levels of Cm-AGA were determined by SDS-PAGE. The protein solution (20 μL) to be tested was mixed with a quarter of the volume of protein loading buffer (5 μL) and then boiled for 10 min. After the boiled samples were briefly centrifuged, they were separated by 6% SDS-PAGE using a Mini-PROTEAN II electrophoresis unit (Bio-Rad Laboratories, Richmond, CA, USA). Protein bands were visualized using Coomassie brilliant blue R-250 (0.25%) staining.

### 2.4. Hydrolysis Reaction of Polysaccharides

Cm-AGA (140 U/mg) was incubated with dissolved agarose (0.5% (*w*/*v*)) in 20 mM Tris-HCl buffer (pH 8.0) at 35 °C (the optimal reaction temperature) for 24 h and sampled. To evaluate the degree of polymerization (DP) of Cm-AGA’s hydrolysates, β-galactosidase was used to cut D-Gal residues [25]. β-galactosidase [26] (BR, *Aspergillus oryzae* extract, Shanghai Yuanye Bio-Technology Co., LTD., Shanghai, China) was added to the reaction mixture and incubated at 55 °C for another 24 h. The reactions were then terminated by boiling the mixture for 15 min. The mixture was then centrifuged at 10,000× *g* for 10 min, and the supernatant was filtered through a 0.22 μm filter for further analysis.

### 2.5. Hydrolysis Reaction of AGO

AGO including agarotriose (A3, purity > 98%), agaropentose (A5, purity > 98%), agaroheptose (A7, purity > 98%), agaronanose (A9, purity > 95%), neoagarobiose (NA2, purity > 98%), neoagarotetraose (NA4, purity > 98%), and neoagarohexaose (NA6, purity > 98%) (Qingdao BZ Oligo Biotech Co., LTD., Qingdao, Shandong, China) were dissolved in Milli-Q water to achieve a final concentration of 5 mg/mL for each. Agarotetraose (A4, purity > 90%) was obtained by separating and purifying enzymatic hydrolysates. The reaction mixtures consisting of 44 μL of purified Cm-AGA (140 U/mg) and 456 μL of substrates (5 mg/mL AGO standards) were incubated at 35 °C for 24 h and sampled. After the reaction was completed, the mixtures were boiled for 15 min and centrifuged at 10,000× *g* for 10 min. The supernatant was filtered through a 0.22 μm filter for further analysis.

### 2.6. Isolation and Purification of Cm-AGA-Produced AOS

To obtain high-concentration products, the oligosaccharide samples were lyophilized and dissolved in Milli-Q water at 10 mg/mL, and then separated through size exclusion chromatography [27] by using a Superdex 30 Increase 10/300 GL column (10 mm × 300 mm, Cytiva, MA, USA). The column was equilibrated and eluted with Milli-Q water at a 0.1 mL/min flow rate. Each fraction (0.2 mL/tube) was collected sequentially and analyzed through high-performance anion exchange chromatography by using the pulsed amperometric detection (HPAEC-PAD) system (ICS-5000A Plus, Thermo Fisher Scientific, Waltham, MA, USA) [28,29,30] to verify AGO purity. The pure oligosaccharide fractions were selected and lyophilized for further characterization.

### 2.7. Product Analysis

D-Gal (purity > 99%), L-AHG (purity ≥ 98%) (Shanghai Yuanye Bio-Technology Co., LTD., Shanghai, China), and AGO standards were diluted to 10 μg/mL. The samples were filtered through 0.22 μm aqueous phase membrane. The samples and standards were tested using HPAEC-PAD. The analysis was performed as follows: The mobile phase consisted of 96% solution containing 100 mM NaOH and 4% solution containing 100 mM NaOH and 500 mM NaAc passed through the Dionex CarboPac PA-200 anion exchange column to equilibrium. The flow rate, column temperature, and sample volume were 0.5 mL/min, 35 °C, and 10 μL, respectively. The detection of four-potential waveform pulse amperes was employed in the experiment. AOS identity and concentration in the samples were determined on the basis of the retention time and peak area of the corresponding peaks.

### 2.8. Molecular Mass Determination

The samples were diluted based on the reducing-sugar content, and the standards D-Gal, L-AHG, and AGO were diluted to 1 μg/mL. The diluted samples were filtered through 0.22 μm aqueous membrane and then detected through ultra-performance liquid chromatography–electrospray ionization–quadrupole–time of flight–mass spectrometry (UPLC-Q-TOF-MS) (ACQUITY UPLC I-Class Plus/Synapt XS, Waters Corporation, Milford, CT, USA) [31,32]. The detection procedures were as follows: Mobile phase A was an acetonitrile solution containing 0.1% (*v*/*v*) ammonia water, and mobile phase B was a mixture of water and methanol at 2:1. Both were passed through the ACQUITY UPLC BEH Amide column to equilibrium. The flow rate, column temperature, and sample volume were 0.3 mL/min, 40 °C, and 2 μL, respectively. Mass spectrometry was performed using an electrospray ionization (ESI) source in a negative ion mode. All mass spectrometry results were processed using MassLynx software (version 4.1) [33].

### 2.9. Multiple Amino Acid Sequence Alignments

Nucleotide sequence similarity of Cm-AGA was compared through a BLAST search [34] (https://blast.ncbi.nlm.nih.gov/Blast.cgi, accessed on 3 January 2023). Cm-AGA and other agarase members of GH96, including AgaA from *Alteromonas agarlyticus* strain GJlB [18], AgaA33 from *Thalassomonas* sp. strain JAMB-A33 [35], AgaD and AgaE from *Thalassomonas* sp. LD5 [36,37], CaLJ96 from *Catenovulum agarivorans* [38], AgaWS5 from *C. sediminis* WS1-A [39], Ce2834 and Ce2835 from *Colwellia echini* A3^T^ [40], were used to perform multiple sequence alignment by using Clustalw (https://www.genome.jp/tools-bin/clustalw, accessed on 5 January 2023) and ESPript 3.0 (https://espript.ibcp.fr/ESPript/ESPript/, accessed on 5 January 2023) services. The Cm-AGA secondary structure was obtained using the NovoPro (https://www.novoprolabs.com/tools/codon-optimization, accessed on 10 May 2023) service.

### 2.10. Protein Structural Prediction and Molecular Docking

The three-dimensional structure of Cm-AGA was predicted using AlphaFold software (version 2.3) [41]. The Cm-AGA protein molecule was visualized using Pymol software (version 2.5.4) [42]. To explore substrate-binding modes, molecular docking was performed using Cm-AGA with linear oligosaccharides of A7, which was generated using ChemDraw software (version 21.0) [43] and Chem3D software (version 21.0) [44]. Molecular docking was conducted using Autodock Vina software (version 5.1.2) [45]. Schematic diagrams of Cm-AGA and A7 interactions were generated using ChimeraX software (version 1.5) [46].

### 2.11. Construction of Mutants and Production of Cm-AGA Mutant Proteins

Plasmid pET-28a(+)/*Cm-aga* containing the *Cm-aga* gene was used as the template. Primers were designed to induce mutations. Asp994 was mutated into Ala, Glu, Arg, and Lys, corresponding to the mutants D994A, D994E, D994K, and D994R, respectively. Glu1129 was mutated into Ala, Asp, Arg, and Lys, corresponding to the mutants E1129A, E1129D, E1129K, and E1129R, respectively. PCR technology was used to construct target gene fragments at Asp994 and Glu1129. After the PCR products were purified by a DNA Gel Extraction Kit (Beyotime, Shanghai, China), they were cloned into the pET-28a(+) vector by homologous recombination (Vazyme, Nanjing, China) to construct the mutant recombinant plasmids. All primers are shown in Table 1. The recombinant plasmids were transferred into *E. coli* JM109. The desired mutations were confirmed by DNA sequencing and the successfully constructed plasmids were transformed into *E. coli* BL21(DE3). The production of multiple mutant proteins of Cm-AGA is the same as that of the wild type mentioned above.

### 2.12. Statistical Analysis

All experimental results are shown as repeated results of three experiments.

## 3. Results

### 3.1. Expression and Purification of Cm-AGA

**Figure 1:**
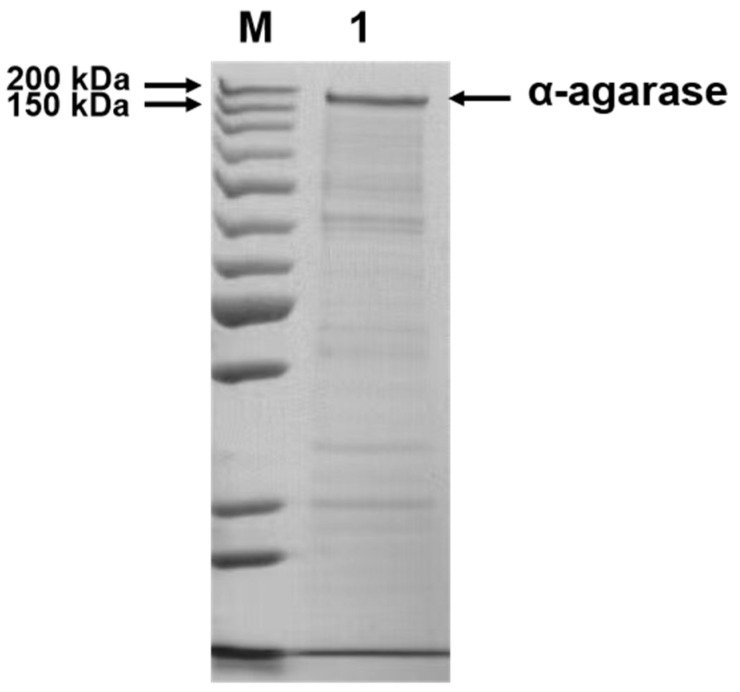
The SDS-PAGE analysis of Cm-AGA. Lane M: molecular weight marker; Lane 1: purified enzyme.

The recombinant plasmid was transferred into *E. coli* BL21(DE3) to produce the recombinant Cm-AGA. The harvested cells were centrifuged, collected, and disintegrated. The crude recombinant enzyme was then purified using nickel affinity chromatography and its molecular weight was estimated as 156.09 kDa [20]. The enzyme was subjected to SDS-PAGE (Figure 1). The purified Cm-AGA exhibited a single, distinct band with a molecular mass of approximately 160 kDa. This was consistent with the molecular weight predicted by DANMAN. This proved that the enzyme was successfully expressed and purified.

### 3.2. Product Analysis Using Agarose as the Substrate

**Figure 2:**
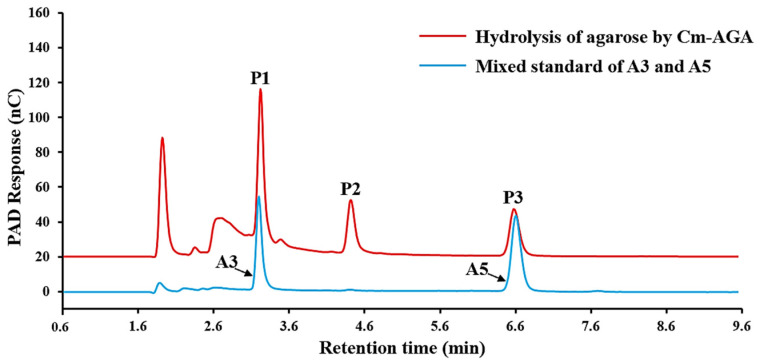
HPAEC-PAD chromatogram of the product from the hydrolysis of agarose by Cm-AGA.

**Figure 3:**
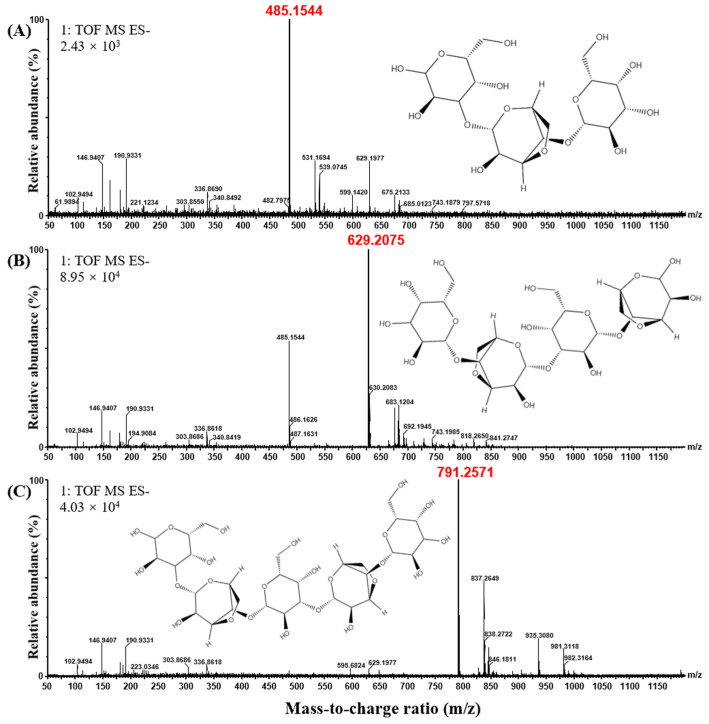
Negative ion UPLC-Q-TOF-MS diagram of the product from the hydrolysis of agarose by Cm-AGA. (**A**) Cm-AGA hydrolysis product P1; (**B**) Cm-AGA hydrolysis product P2; (**C**) Cm-AGA hydrolysis product P3.

Cm-AGA was incubated with agarose at 35 °C for 24 h. The product was analyzed using HPAEC-PAD. Three distinct peaks P1, P2, and P3 were detected at the retention time of 3.3 min, 4.5 min, and 6.6 min, respectively (Figure 2). The odd-numbered AOSs (A3, A5) were used as standards. According to the comparison with the positions of A3 and A5 standards, it is determined that P1 and P3 correspond to A3 and A5, respectively, and it is inferred that the peak P2 in the middle corresponds to A4. To further identify the DPs of the product, the molecular weight of the product was determined by UPLC-Q-TOF-MS, and the results are shown in Figure 3. By analyzing these three products, underived AOSs were eluted through gradient elution by using the BEH amide column. AOSs were optimally separated on the column in the ascending order of DPs, yielding molecular weights of 485 Da (P1), 629 Da (P2), and 791 Da (P3), respectively. The molecular weight values of AGOs with different DPs are shown in Table 2. The mass spectrometry data (*m*/*z*) of AGOs in positive and negative ion modes are shown in Table 3. This indicates that P1, P2, and P3 are AOSs with the DPs of 3, 4, and 5 in each. A3, A4, and A5 were the main products, respectively.

**Figure 4:**
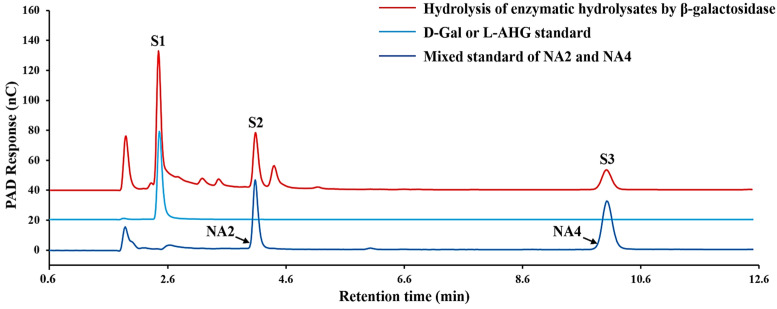
HPAEC-PAD chromatogram of the product from the rehydrolysis of Cm-AGA enzymatic hydrolysates by β-galactosidase.

**Figure 5:**
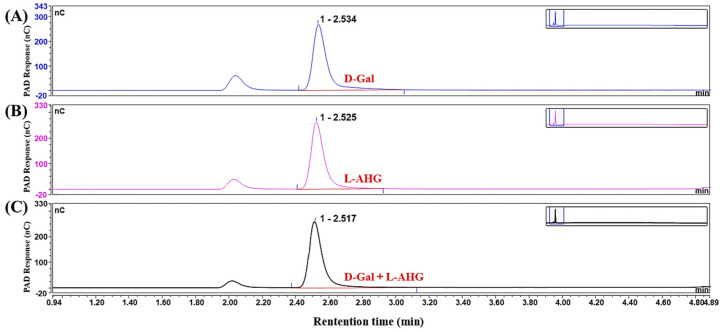
HPAEC-PAD analysis of galactose standards. (**A**) β-D-galactose (D-Gal) standard. (**B**) 3,6-anhydro-α-l-galactose (L-AHG) standard. (**C**) D-Gal and L-AHG mixed standards.

**Figure 6:**
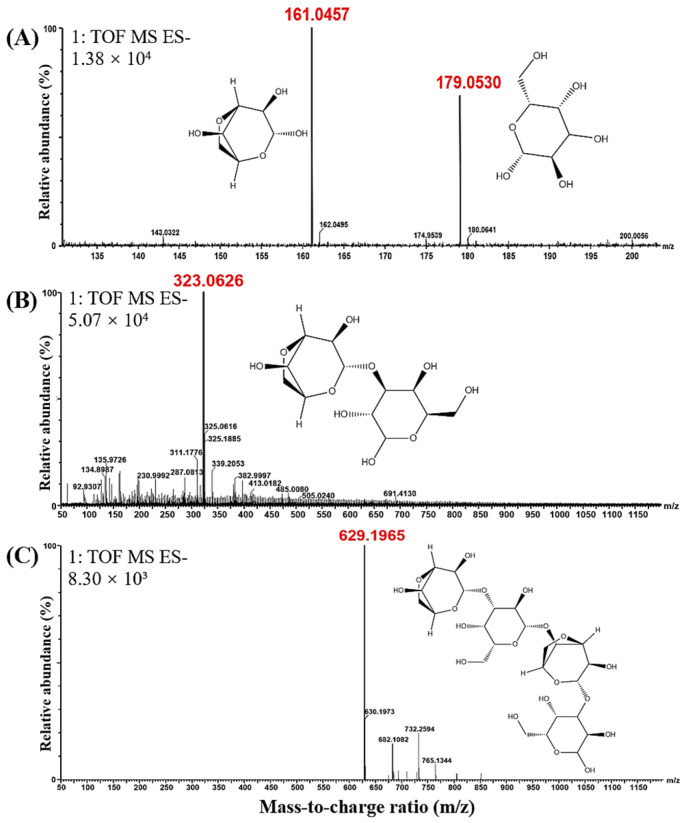
Negative ion UPLC-Q-TOF-MS diagram of the product from the rehydrolysis of Cm-AGA enzymatic hydrolysates by β-galactosidase. (**A**) Final hydrolysate S1. (**B**) Final hydrolysate S2. (**C**) Final hydrolysate S3.

β-galactosidase is a glycoside exonuclease [25] that cleaves D-galactose residues linked by β-1-4-glycoside bonds at the non-reducing end [47]. And β-galactosidase was used to further degrade the enzymatic hydrolysates. The HPAEC-PAD analysis revealed that β-galactosidase degraded products P1, P2, and P3 to S1, S2, and S3, respectively (Figure 4). It is noted that the retention times of the peaks corresponding to D-Gal and L-AHG standards in this study were consistent (Figure 5), suggesting similar molecular weights and structures. Compared with the AGO standards, the significantly increased peaks of the obtained products corresponded to D-Gal and L-AHG, NA2, and NA4, and the corresponding molecular weights were 161/179, 323, and 629 Da (Figure 6).

Based on the hydrolysis mechanism of β-galactosidase [25,47], wherein the enzyme specifically cleaves the first β-1,4 linkage at the non-reducing end of oligosaccharides, we predicted that A3 would be further degraded into D-Gal and NA2, A4 would be further degraded into D-Gal and NA3, and A5 would be further degraded into D-Gal and NA4. However, different from these predictions, no new peak (NA3 peak) was observed within the retention time range of 4.1 min to 10.0 min in the HPAEC-PAD analysis, possibly due to the spontaneous release of L-AHG at the reducing end of NA3 and the simultaneous production of NA2. The reducing end of agar-derived oligosaccharides spontaneously sheds unstable L-AHG molecules [36,40]. Combined with the above results, it is reasonable to assume that the odd-numbered AOSs A3 and A5 in the enzymatic hydrolysates of Cm-AGA are derived from A4 and A6, respectively. Therefore, the main products of the hydrolysis of agarose by Cm-AGA are A4 and A6, which is consistent with the products reported for other α-agarases such as AgaA [18] and AgaE [37].

### 3.3. Identification of the Cm-AGA Hydrolysis Pattern

**Figure 7:**
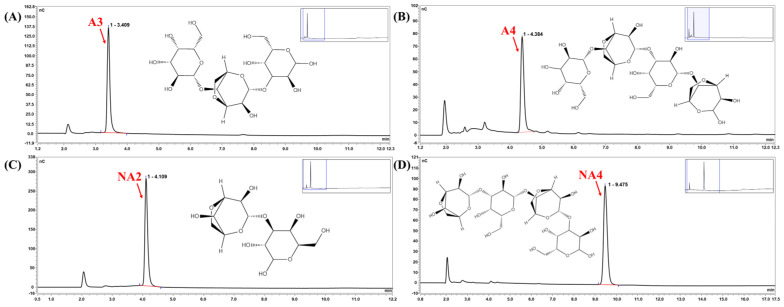
HPAEC-PAD analysis of the products obtained from the degradation of A3 (**A**), A4 (**B**), NA2 (**C**), and NA4 (**D**) by Cm-AGA.

To further demonstrate that A4 and A6 products can yield A3 and A5, respectively, Cm-AGA was used to degrade the AGOs (A3, A4, A5, A7, A9, NA2, NA4, and NA6). After Cm-AGA was incubated with A3, A4, NA2, and NA4 at 35 °C for 24 h, only one peak was detected for each AGO (Figure 7). No additional peaks were generated in the mass spectrometry results, demonstrating that Cm-AGA cannot act on the A3, A4, NA2, and NA4 substrates. The results are similar to those of AgaD [36], CaLJ96 [38], AgaA [18], and AgaA33 [35]. On analyzing similar nucleotide sequences by using BLAST, we noted that Cm-AGA shares a sequence identity of 95.21% with AgaD and 89.79% with AgaA. The analysis of different α-agarase hydrolysates studied is presented in Table 4. AgaA, Aga33, CaLJ96, AgaWS5, AgaD, and AgaE can degrade agaroses to produce even-numbered oligosaccharides; A4 was the main product. Notably, AgaA, AgaD, and AgaWS5 can also simultaneously be used to produce odd-numbered oligosaccharides through agarose degradation. Specifically, native AgaA demonstrated activities for both α-agarase and β-galactosidase. By breaking down agarose, this agarase complex generated a mixture of AOSs belonging to the A4 and A3 series. Of them, the A3 series contained oligosaccharides with an odd number of galactose residues. AgaD breaks down agarose to yield the predominant end product, A4, which is rapidly degraded into A3 with the G-A-G sequence (G, D-Gal; A, L-AHG) under strong alkaline conditions. Similarly, the main hydrolysis product of AgaWS5 is A4. Under alkaline conditions, the L-AHG residue of A4 (G-A-G-A) at the reducing end was readily cleaved, which resulted in the A3 formation (G-A-G). In summary, the enzymatic hydrolysates of Cm-AGA are indeed A4 and A6, and this enzyme cannot act on monosaccharides of tetrasaccharides and less.

**Figure 8:**
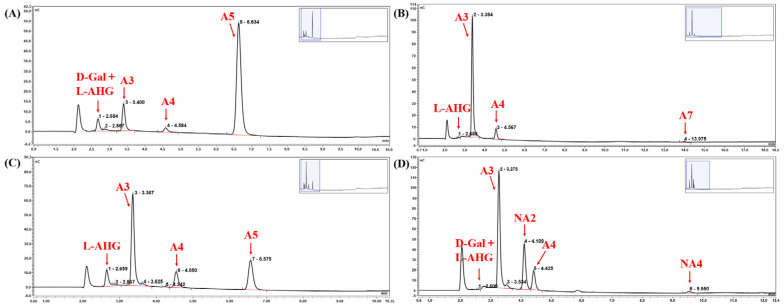
HPAEC-PAD analysis of the products obtained from the degradation of A5 (**A**), A7 (**B**), A9 (**C**), and NA6 (**D**) by Cm-AGA.

**Figure 9:**
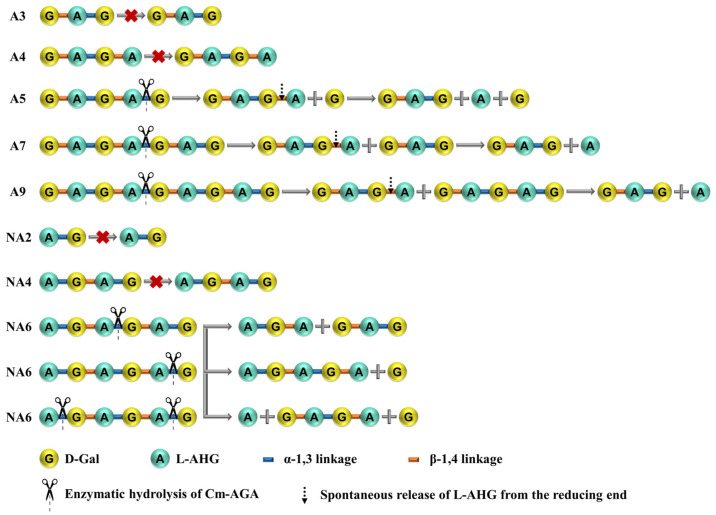
Reaction scheme of the GH96 α-agarase Cm-AGA hydrolyzing AGOs (A3, A4, A5, A7, A9, NA2, NA4, and NA6).

The HPAEC-PAD analysis results of the products obtained through Cm-AGA-mediated A5 degradation are presented in Figure 8A. A5 accounted for the majority of the spectrum, indicating that Cm-AGA degraded only a small portion of A5. Cm-AGA sheared from the non-reducing end of oligosaccharides [48] and hydrolyzed the second α-1,3 linkage of A5 to generate A4 and D-Gal, where A4 further yielded A3 because the reducing end L-AHG is unstable [40]. Accordingly, Cm-AGA-mediated AGO hydrolysis is illustrated by the reaction scheme in Figure 9. The HPAEC-PAD analysis results for the products of Cm-AGA-mediated degradation of long-chain AGO are presented in Figure 8B,C. The second α-1,3 linkage of A7 were hydrolyzed to generate A3 and A4 and those of A9 were hydrolyzed to produce A4 and A5. As with A5, A4 obtained from A7 and A9 also further generated L-AHG and A3. Therefore, pentasaccharides were the minimum length of the substrate degraded by Cm-AGA. Interestingly, the products of Cm-AGA-mediated NA6 hydrolysis were relatively complex (Figure 8D), including A3, NA2, A4, NA3, and NA4, which differ from the results of Duleepa Pathiraja et al. [40].

Based on the aforementioned results, Cm-AGA is speculated to exhibit three hydrolysis modes for NA6. The first mode involves the hydrolysis of the second α-1,3 linkage of NA6, forming NA3 and A3, and NA3 releases one L-AHG moiety to obtain NA2. The second mode involves the hydrolysis of the third α-1,3 linkage of NA6, generating NA5 and D-Gal, and NA5 releases L-AHG to obtain NA4. The third mode involves the hydrolysis of the first and third α-1,3 linkages of NA6, resulting in A4. Cm-AGA is inferred to possess the activity of exo-cutting α-1,3 linkages for NAOS with a DP greater than 4. According to the peak appearance of each product after Cm-AGA hydrolysis of NA6 in the chromatogram, it was found that the peaks of A3 and NA2 were relatively higher, indicating that the hydrolysis of NA6 by Cm-AGA is mainly in the first mode.

### 3.4. Multiple Amino Acid Sequence Alignments and Site-Directed Mutation Verification

**Figure 10:**
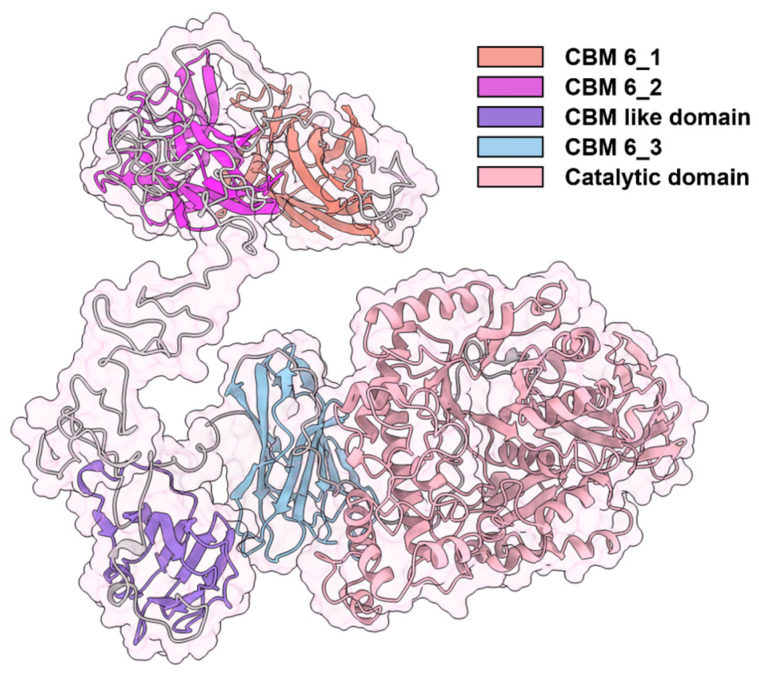
The 3D structure model of Cm-AGA predicted by AlphaFold 2.3.

The relationship between the structure and function of Cm-AGA is currently unknown. To explore the binding sites and catalytic processes for Cm-AGA, the hydrolysis mechanism and structure–activity relationship of this enzyme need to be further evaluated. AlphaFold was used to predict the three-dimensional structure of Cm-AGA (Figure 10). According to the 3D structure model, Cm-AGA has three carbohydrate-binding module 6 (CBM6) domains for substrate binding (CBM_1, residue 1–136; CBM_2, 182–320; CBM_3, 667–802), one thrombospondin type 3 (TSP-3) domain for calcium ion binding, and one CBM-like domain consisting of nine antiparallel β layers. A typical catalytic domain structure, which contained the (α/β)_6/7_ barrel structure and catalytic grooves similar to those found in α-amylases in the GH13 family, was noted [49]. Therefore, this region is considered as the catalytic domain.

**Figure 11:**
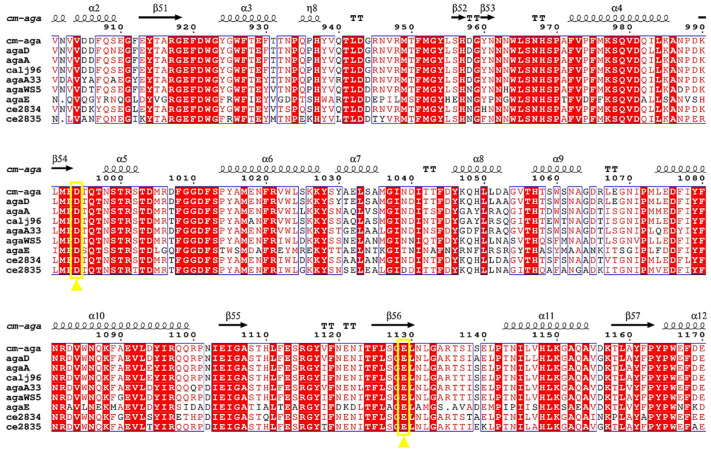
Sequence alignment of sites 900 to 1170 of α-agarase gene Cm-AGA with agaD, agaA, calj96, agaA33, agaWS5, agaE, ce2834, and ce2835. (The secondary structural components of Cm-AGA are marked above the sequence. Conservative residues are represented by red squares with white letters, while similar residues are represented by red letters. The key residues Asp994 and Glu1129 are marked with yellow boxes and triangles.)

The active sites of glycoside hydrolases require two highly conserved amino acids [50], typically Glu and Asp (5.5 Å interval), with both of them proposed to provide acid/base and nucleophilic assistance [51]. By conducting site-directed mutagenesis studies on the conserved carboxyl amino acids of GH96, Xu et al. [37] revealed two catalytically essential amino acids for α-agarase AgaE, namely Asp779 and Asp781. Mutation at Asp779 or Asp781 led to a drastic loss of enzymatic activity, and the site Asp781 is involved in Na binding. Allouch et al. [52] reported the structures of β-agarases A and B from *Zobellia galactanivorans* Dsij. These enzymes possess a deep active site channel harboring the catalytic machinery, namely the nucleophilic residues Glu147 and Glu184 and the acid/base residues Glu152 and Glu189. Asp271 was identified as a crucial residue for substrate interaction with the surface-binding site in β-agarase from *Z. galactanivorans* [53]. Thus, we identified Asp and Glu residues that played catalytic roles in the absolutely conserved region through the multiple sequence alignment of nine α-agarase genes (Figure 11). Asp994 and Glu1129 could serve as catalytic residues of Cm-AGA, with a distance of 5.79 Å existing between them. Asp994 serves as a general base that facilitates the nucleophilic attack of water on the glycosidic bond, while Glu1129 serves as a general acid that simultaneously donates a proton to the aglycone leaving group [54].

**Figure 12:**
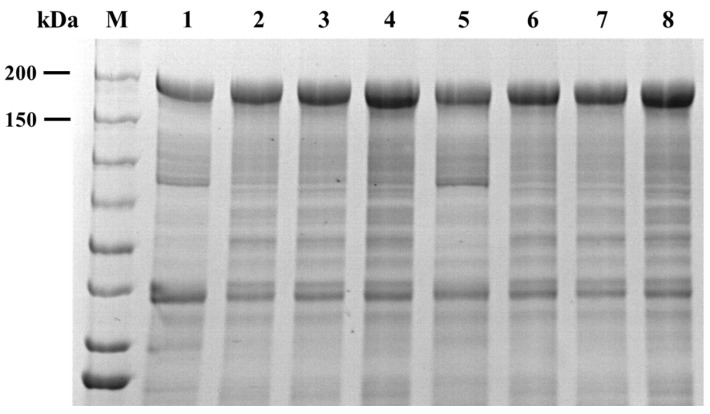
The SDS-PAGE analysis of Cm-AGA mutants. Lane M: molecular weight marker; Lane 1, Lane 2, Lane 3, and Lane 4 correspond to the mutants D994A, D994E, D994K, and D994R, respectively. Lane 5, Lane 6, Lane 7, and Lane 8 correspond to the mutants E1129A, E1129D, E1129K, and E1129R, respectively.

In order to further determine the catalytic site performance of Asp994 and Glu1129, site-directed mutation verification experiments were conducted. After fermentation, the crude enzyme solution was collected and purified and then combined with SDS-PAGE to further verify the expression of Cm-AGA mutant. As shown in Figure 12, the molecular weight of the mutant was consistent with that of the wild type, approaching 156.09 kDa. The enzyme activity of these mutants was found to be inactivated by the DNS method. It can be seen that Asp994 and Glu1129 are the exact catalytic sites of Cm-AGA.

### 3.5. Molecular Docking

**Figure 13:**
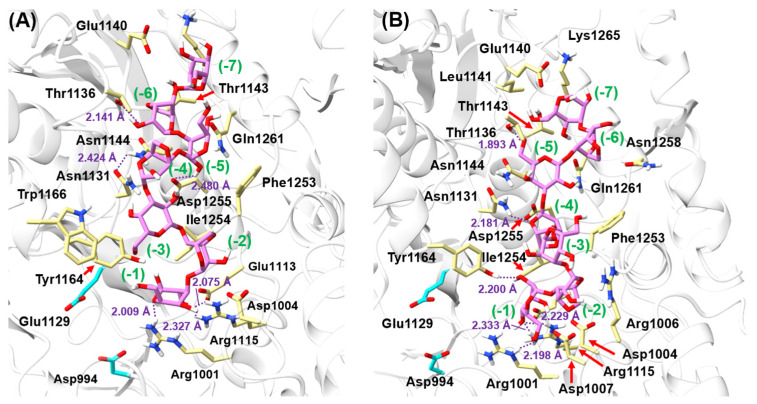
The binding conformations of A7 ligand in Cm-AGA. (**A**) The reducing end of the A7 chain is close to the Asp994 and Glu1129. (**B**) The non-reducing end of the A7 chain is close to the Asp994 and Glu1129. The A7 ligand is represented with purple sticks, and the residues contributing strong interactions with the ligand are represented with yellow sticks. The catalytic dyad in Cm-AGA are presented with cyan sticks.

While the protein sequence remained identical, some structural rearrangement occurred on the binding A7 ligand. There are two different substrate-binding modes: in one mode, the reducing end of the A7 chain is close to the two catalytic residues, and in the other mode, the non-reducing end of the A7 chain is close to the two catalytic residues (Figure 13). Docking simulations revealed that five (Gly1133, Thr1143, Asn1144, Ile1254, and Lys1265) overlapping amino acids had hydrophobic interactions with A7, and nine (Asp1004, Glu1113, Arg1115, Asn1131, Thr1136, Glu1140, Tyr1164, Asp1255, and Gln1261) overlapping amino acids formed hydrogen bonds with A7 in the substrate-binding groove. One substrate-binding mode demonstrated that Arg1001, Trp1166, and Gly1252 bind through hydrophobic interaction force with A7. Similarly, the other substrate-binding mode showed that Arg1006 and Asn1258 can have hydrophobic interactions with A7, and Arg1001, Asp1007, and Leu1141 form hydrogen bonds with A7. In both binding modes, the aromatic amino acids Tyr1164 and Phe1253 may offer π–π stacking interactions on both sides of A7 near the catalytic cavity.

## 4. Discussion

Cm-AGA hydrolyzed agarose, leading to A4 and A6 formation. Moreover, the reducing end of both AOS spontaneously released unstable L-AHG molecules, which were further transformed to yield A3 and A5. Cm-AGA exerted its activity on AGO with a DP greater than 4, while it could not act on α-1,3-glucoside bonds in A3, A4, NA2, and NA4. The catalytic domain of Cm-AGA includes an (α/β)_6/7_ TIM barrel, which is similar to that of GH13 amylase. Within a groove located at a distance of 5.79 Å, two conserved residues, Asp994 and Glu1129, are proposed to act as catalytic residues, which have been identified to be correct by directed mutagenesis experiments. Through molecular docking analysis, A7 was noted to stably bind in the catalytic groove, and residues Tyr1164 and Phe1253 located on the two sides of the catalytic cavity could provide hydrophobic and electrostatic forces to bind with substrates. This study investigated the substrate-binding mode of Cm-AGA to understand the relationships between substrates and its structure, thereby laying a foundation for the targeted production of AOS and the engineering of enzymes to improve catalytic performance.

## Figures and Tables

**Table 1:** Primers used for site-directed mutagenesis of Cm-AGA.

Mutant	Primer Sequence (5′-3′) ^1^
CM_D994A_F1	ATAAACTGATGTTT***GCC***ACGCAGACAAATAGCACGAGAAG
CM_D994A_F2	GCGCGTAATCTGCTGCTTG
CM_D994A_F3	CCCACGCCGAAACAAGCG
CM_D994A_R3	CAAACATCAGTTTATCCGGGTTCGC
CM_D994E_F1	ATAAACTGATGTTT***GAA***ACGCAGACAAATAGCACGAGAAG
CM_D994E_F2	GCGCGTAATCTGCTGCTTG
CM_D994E_F3	CCCACGCCGAAACAAGCG
CM_D994E_R3	CAAACATCAGTTTATCCGGGTTCGC
CM_D994K_F1	GATAAACTGATGTTT***AAA***ACGCAGACAAATAGCACGAGAAG
CM_D994K_F2	GCGCGTAATCTGCTGCTTG
CM_D994K_F3	CCCACGCCGAAACAAGCG
CM_D994K_R3	AAACATCAGTTTATCCGGGTTCGCC
CM_D994R_F1	GATAAACTGATGTTT***CGC***ACGCAGACAAATAGCACGAGAAG
CM_D994R_F2	GCGCGTAATCTGCTGCTTG
CM_D994R_F3	CCCACGCCGAAACAAGCG
CM_D994R_R3	AAACATCAGTTTATCCGGGTTCGCC
CM_E1129A_F1	CATTCCTGAGCGGC***GCA***CTGAATCTGGGCGCAAGAACATC
CM_E1129A_F2	GCGCGTAATCTGCTGCTTG
CM_E1129A_F3	CCCACGCCGAAACAAGCG
CM_E1129A_R3	CGCCGCTCAGGAATGTAATGTTC
CM_E1129D_F1	CATTCCTGAGCGGC***GAC***CTGAATCTGGGCGCAAGAACATC
CM_E1129D_F2	GCGCGTAATCTGCTGCTTG
CM_E1129D_F3	CCCACGCCGAAACAAGCG
CM_E1129D_R3	CGCCGCTCAGGAATGTAATGTTC
CM_E1129K_F1	ACATTCCTGAGCGGC***AAG***CTGAATCTGGGCGCAAG
CM_E1129K_F2	GCGCGTAATCTGCTGCTTG
CM_E1129K_F3	CCCACGCCGAAACAAGCG
CM_E1129K_R3	GCCGCTCAGGAATGTAATGTTCTC
CM_E1129R_F1	ACATTCCTGAGCGGC***CGG***CTGAATCTGGGCGCAAG
CM_E1129R_F2	GCGCGTAATCTGCTGCTTG
CM_E1129R_F3	CCCACGCCGAAACAAGCG
CM_E1129R_R3	GCCGCTCAGGAATGTAATGTTCTC
CM_R1 ^2^	CAAGCAGCAGATTACGCGC
CM_R2 ^3^	CGCTTGTTTCGGCGTGGG

^1^ The underline indicates the base sequence corresponding to the mutation site. ^2^ All of the Cm-AGA mutant DNA fragments are designed using the CM_R1 reverse primer. ^3^ All of the Cm-AGA mutant DNA fragments are designed using the CM_R2 reverse primer.

**Table 2:** Molecular weight of oligosaccharides with different degrees of polymerization.

Oligosaccharide Types	Molecular Weight (Da)
3,6-anhydro-L-galactose (L-AHG)	162.14
D-Galactose (D-Gal)	180.16
Agarobiose (A2), Neoagarobiose (NA2)	324.28
Agarotriose (A3), Neoagarotriose (NA3)	486.42
Agarotetraose (A4), Neoagarotetraose (NA4)	630.00
Agaropentose (A5), Neoagaropentose (NA5)	792.69
Agarohexaose (A6), Neoagarohexaose (NA6)	936.00
Agaroheptose (A7), Neoagaroheptose (NA7)	1098.95
Agarooctaose (A8), Neoagarooctaose (NA8)	1281.00
Agaronanose (A9), Neoagaronanose (NA9)	1405.22

**Table 3:** Ions identified for the AGOs in both positive- and negative-ion mode of ESI-MS (m/z).

Ion Mode	Ions	Degree of Polymerization for Oligosaccharides			
		3	5	7	9
ESI+	[M + H]^+^	487.2	793.3	1099.4	1405.4
ESI−	[M − H]^−^	485.1 *	791.3 *	1097.4 *	1403.6 *

* The base peak ion.

**Table 4:** The analysis of different α-agarase enzymatic hydrolysates studied.

Protein	Bacterial Species	GH Family	Substrates Used in the Study	Main Products	Remarks
AgaA	*Alteromonas agarlyticus* Strain GJlB	GH96	Agarose	A4, A6	Does not act on A4 and A6, possess agarase and β-galactosidase activities
Aga33	*Thalassomonas* sp. Strain JAMB-A33	GH96	Agarose, A4, A6, NA4, NA6	A4	Acts on A6 and NA6, but cannot hydrolyze tetrasaccharides
AgaD	*Thalassomonas* sp. LD5	GH96	Agarose	A4	Under alkaline conditions, A4 degrade into A3 with a G-A-G arrangement
			A4, A6, A8, A10		Does not act on A2 and A4, and the minimum-length substrate is A6
CaLJ96	*Catenovulum agarivorans*	GH96	Agarose, A3, A5	A4	Does not act on A2, A3, or A4, and the minimum-length substrate is A5
AgaWS5	*Catenovulum sediminis* WS1-A	GH96	Agarose	A4	Under alkaline circumstances, L-AHG residues at the reducing end of A4 (G-A-G-A) were cleaved to form A3 (G-A-G)
AgaE	*Thalassomonas* sp. LD5	GH96	Agarose	A4, A6	None
Ce2834, Ce2835	*Colwellia echini* A3^T^	GH96	Agarose, NA4, NA6	A4, A6	None
Cm-AGA	*Catenovulum maritimum* STB14	GH96	Agarose	A2, A4	None

## Data Availability

The original contributions presented in the study are included in the article, further inquiries can be directed to the corresponding author.
